# Cardiac and mitochondrial function in HIV-uninfected fetuses exposed to antiretroviral treatment

**DOI:** 10.1371/journal.pone.0213279

**Published:** 2019-03-04

**Authors:** Laura García-Otero, Marta López, Mariona Guitart-Mampel, Constanza Morén, Anna Goncé, Carol Esteve, Laura Salazar, Olga Gómez, Josep María Martínez, Berta Torres, Sergi César, Glòria Garrabou, Fàtima Crispi, Eduard Gratacós

**Affiliations:** 1 Fetal i+D Fetal Medicine Research Center, BCNatal—Barcelona Center for Maternal-Fetal and Neonatal Medicine (Hospital Clínic and Hospital Sant Joan de Deu), Institut Clinic de Ginecologia, Obstetricia i Neonatologia (ICGON), Institut d’Investigacions Biomèdiques August Pi i Sunyer (IDIBAPS), Universitat de Barcelona and Centre for Biomedical Research on Rare Diseases (CIBER-ER), Barcelona, Spain; 2 Muscle Research and Mitochondrial Function Laboratory, Cellex IDIBAPS, Faculty of Medicine and Health Sciences-University of Barcelona, Internal Medicine Service-Hospital Clínic of Barcelona (Barcelona, Spain) and CIBER-ER; 3 Infectious Diseases Department, Hospital Clínic, Fundació Clínic per a la Recerca Biomèdica (FCRB), Barcelona, Spain; 4 Department of Pediatric Cardiology, Hospital Sant Joan de Déu Barcelona, University of Barcelona, Barcelona, Spain; Azienda Ospedaliera Universitaria di Perugia, ITALY

## Abstract

**Background:**

Mitochondrial toxicity related to maternal combined antiretroviral treatment (cART) may have an impact on the heart of HIV-exposed uninfected (HEU) fetuses. Our objective was to evaluate fetal cardiovascular and mitochondrial biomarkers in HIV pregnancies.

**Methods:**

Prospective cohort including 47 HIV-infected and 47 non HIV-infected pregnancies. Fetal echocardiography was performed at 26–32 weeks of pregnancy. Umbilical cord blood and placental tissue were collected to study mitochondrial DNA content (mtDNA) (ratio 12SrRNA/RNAseP) and mitochondrial function (cytochrome c oxidase, COX, enzymatic activity) normalized by mitochondrial content (citrate synthase, CS).

**Results:**

HEU fetuses showed hypertrophic hearts (left myocardial wall thickness: HIV mean 3.21 mm (SD 0.81) vs. non-HIV 2.72 (0.42), p = 0.012), with signs of systolic and diastolic dysfunction (isovolumic relaxation time: HIV 52.2 ms (8.85) vs. non-HIV 42.5 ms (7.30); p<0.001). Cord blood mitochondrial content was significantly increased in HIV-exposed fetuses (CS activity: HIV 82.9 nmol/min.mg of protein (SD 40.5) vs. non-HIV 56.7 nmol/min.mg of protein (28.4); p = 0.007), with no differences in mtDNA content and COX activity. Both myocardial and mitochondrial mass parameters were significantly associated with zidovudine exposure.

**Conclusions:**

HEU fetuses showed signs of increased myocardial and mitochondrial mass associated with maternal zidovudine treatment, suggesting a fetal adaptive response to cART toxicity.

## Introduction

Perinatal transmission of Human Immunodeficiency Virus (HIV) is mainly prevented by combined antiretroviral treatment (cART) during pregnancy [1]. In 2016, about 76% [60–88%] of pregnant women living with HIV worldwide had access to cART [1–4]. As a result, the number of HIV-exposed uninfected (HEU) children has been on the rise, with health conditions presented, even if mild, having a potentially noteworthy public health impact [5]. Indeed, it is well known that an adverse prenatal environment during the critical period of *in utero* fetal development might have long lasting consequences on health [6].

HEU children are mainly considered a healthy population, although recent studies have raised concerns regarding their cardiovascular health. Several studies have demonstrated significant changes in the cardiovascular structure and function of HEU offspring from fetal life to adolescence [7–12]. A recent study by our group suggested that fetal cardiac remodeling observed in HIV-infected pregnancies was associated with maternal use of zidovudine [11]. However, the specific mechanism of cardiac remodeling observed in HEU offspring remains to be elucidated. Mitochondrial toxicity related to HIV or cART use during pregnancy may be a potential pathophysiological pathway responsible for these cardiovascular changes.

HIV itself is known to cause mitochondrial DNA (mtDNA) depletion as well as mitochondrial respiratory chain (MRC) disturbances in HIV patients who have never received cART [13]. Moreover, mitochondrial toxicity derived from cART has been widely described in adults, especially as it relates to nucleoside reverse transcriptase inhibitors (NRTIs), which are known to inhibit mtDNA polymerase gamma [14] promoting mitochondrial dysfunction [15] responsible for a wide range of clinical manifestations including myocardiopathy in children and adults [16,17]. During pregnancy, the mitochondrial effects of HIV infection and NRTI exposure have been scarcely assessed with conflicting results [18–20] reporting decreased [21] or increased mtDNA content [20] in HEU children with *in utero* cART exposure and neonatal zidovudine prophylaxis [21–25]. However, none of these studies have investigated the association between the mitochondrial findings and fetal cardiac changes nor have they assessed the role of different cART regimens on the fetal cardiac remodeling mechanism.

The objective of the present study was to evaluate cardiovascular and mitochondrial biomarkers in HEU fetuses from HIV pregnancies under cART as compared to non-HIV-infected pregnancies. Therefore, we designed a cohort of HIV-exposed and HIV-unexposed pregnancies undergoing fetal echocardiography and we determined cardiac and mitochondrial biomarkers in cord blood and placenta.

## Methods

### Study design and population

A prospective cohort study was performed in 47 HEU fetuses from HIV-infected pregnant women under cART followed in the Maternal—Fetal Medicine Department at BCNatal (Hospital Clínic and Hospital Sant Joan de Deu) in Barcelona (Spain) from December 2014 to March 2017. The unexposed group included 47 consecutively recruited non-HIV-infected pregnancies from the same Department accepting to participate. The unexposed group was frequency paired (1:1) with HIV-infected pregnancies by gestational age at fetal echocardiography (± 1 week). Exclusion criteria included multiple pregnancies, diagnosis of fetal malformations or chromosomal abnormalities, delivery before 24 weeks of gestational age as well as perinatal transmission of HIV or familiar history of mitochondrial disease. All individuals were informed, and signed written consent was obtained for inclusion in this study. The study protocol was approved by the Ethical Committee of our hospital (Comité Ético de Investigación del Hospital Clínic de Barcelona (CEIC)) in accordance with the Declaration of Helsinki guidelines. Approval number HCB/2014/0401 ER-01.

The same study protocol was applied in both groups including collection of baseline and perinatal characteristics, third trimester fetal echocardiography and collection of biological samples at delivery (umbilical cord blood and placenta).

### Collection of maternal, perinatal and immunovirological characteristics

Maternal demographic and perinatal characteristics were collected by personal interview or review of medical records. Low socioeconomic status was considered when a patient was illiterate or had a primary educational level. Maternal comorbidity was defined as the presence of chronic hypertension [26], pregestational diabetes [27] or autoimmune disorder [28]. Preeclampsia was defined as new onset of hypertension (systolic pressure > 140 mmHg and/or diastolic pressure > 90 mmHg) together with more than 300 mg proteins in 24 h urine [29]. Preterm delivery was defined as delivery before 37 weeks of gestation and small for gestational age by birth weight below 10^th^ centile according to local standards [30]. Perinatal mortality was defined as intrauterine fetal death after 22 weeks of pregnancy or neonatal death within the first 28 days of life [31]. Major neonatal morbidity was defined by the presence of bronchopulmonary dysplasia, necrotizing enterocolitis, intraventricular hemorrhage, periventricular leukomalacia, retinopathy, persistent ductus arteriosus, or sepsis.

The immunovirological and therapeutic parameters of HIV-infected women were also recorded including mode of transmission, presence of previous opportunistic infections, diagnosis of HIV during pregnancy and months of HIV infection at delivery. The CD4 T-cell count was assessed by flow cytometry and plasmatic viral load by HIV-RNA copy quantification (Amplicor HIV Monitor; Roche Diagnostic Systems, Branchburg, New Jersey, USA) during pregnancy. Hemoglobin levels were recorded in the third-trimester blood test to detect potential maternal anemia. Antiretroviral therapy was administered to all HIV-infected pregnant women following local and international guidelines [32,33]. The recommended combinations included two NRTI with either a non-nucleoside reverse-transcriptase inhibitor (NNRTI) or one or more protease inhibitors (PI) or other antiretroviral drugs such as integrase inhibitors (INI). The type and duration of cART before and during pregnancy were also recorded.

### Fetal echocardiography

Fetal ultrasonographic examination was performed in all pregnancies at 26–32 weeks of gestation using a Siemens Sonoline Antares (Siemens Medical Systems, Malvern, PA, USA) with 6–4- MHz linear curved-array and 2–10 MHz phased-array probes, including evaluation of estimated fetal weight, feto-placental Doppler and echocardiography. Ultrasounds were performed by maternal—fetal medicine specialists skilled in fetal echocardiography who were blinded to the cART regimen but not to the HIV status. Gestational age was calculated according to first trimester crown-rump length [34]. Fetal weight was estimated following the Hadlock formula [35]. Feto-placental Doppler evaluation included the calculation of cerebroplacental ratio [36] as a surrogate of placental insufficiency.

Fetal echocardiography included a comprehensive morphometric and functional assessment by two-dimensional, M-mode, conventional and tissue Doppler following a standard protocol. The cardiothoracic ratio was calculated as heart area/thoracic area [37]. Pericardial effusion was evaluated at the midventricular level from a transverse four-chamber view and considered present when exceeding 2 mm [38]. Myocardial wall thicknesses were measured from a 2D transverse four-chamber view at end-systole [39]. The left ejection fraction was obtained from a transverse four-chamber view by M-mode using the Teichholz‘s formula [40]. Mitral and tricuspid ring displacement were assessed by M-mode from an apical or basal four-chamber view [41]. Systolic (S’) and early diastolic (E’) annular peak velocities were obtained by spectral real time tissue Doppler at tricuspid and lateral mitral annuli [42]. Left isovolumic and ejection times were obtained placing the Doppler sample volume on the medial wall of the ascending aorta, including the aortic and mitral valve. Valvular clicks in the Doppler wave were used as landmarks. Isovolumic contraction time (ICT) was measured from the closure of the mitral valve to the opening of the aortic valve. Ejection time (ET) was measured from the opening to the closure of the aortic valve. Isovolumic relaxation time (IRT) was measured from the closure of the aortic valve to the opening of the mitral valve [43,44]. Myocardial performance index (MPI) was calculated as follows: (ICT + IRT)/ET [44].

### Cord blood and placental samples collection

Fetal umbilical cord blood was collected in EDTA tubes immediately after delivery. Plasma was separated by centrifugation at 1500 rcf for 15 min at room temperature and immediately stored at -80°C until further experimental analysis. In addition, cord blood mononuclear cells (CBMC) were isolated by Ficoll density gradient centrifugation, divided into aliquots and stored at -80°C until mitochondrial analysis. An estimated platelet count < 25 per lymphocyte/monocyte suggested negligible platelet contamination for the analysis of mitochondrial DNA (mtDNA) content [45]

Placental samples were immediately collected after delivery. Tissue samples of villous parenchyma (1–2 cm^3^) were selected from three different sites of three lobules (free of visible infarction, calcification, hematoma or damage) and immediately frozen at −80°C.

Mitochondrial studies were performed in CBMC and placental homogenates (5% w/v in mannitol buffer) [46] following quantification of protein content using the bybicinchoninic acid colorimetric assay (Thermo Scientific assay kit Prod #23225, Waltham, MA, USA) to normalize experimental measures [47].

### Cord blood and placental biomarkers analysis

Cord blood plasmatic levels of B-type Natriuretic Peptide (BNP) were measured using the Siemens ADVIA Centaur BNP assay [48].

Mitochondrial toxicity was evaluated in CBMC and placental homogenates including relative content of mtDNA, cytochrome c oxidase (COX) and citrate synthase (CS) enzymatic activities. Total DNA was obtained by standard phenol-chloroform extraction procedure. A fragment of the mitochondrial-encoded *12SrRNA* gene and the nuclear-encoded *RNAse P* gene were simultaneously amplified in triplicate by quantitative multiplex rtPCR using AbiPrism 7900 system from Applied Biosystems [49]. The relative content of mtDNA was expressed as the ratio between mitochondrial to nuclear DNA amount (12SrRNA mtDNA/ RNAseP nDNA content) as absolute values and as relative values normalized to mitochondrial mass through citrate synthase activity.

Spectrophotometric measurement of COX [EC 1.14.99.1] enzymatic activity was performed according to standardized international protocols [50] as well as CS activity [EC 4.1.3.7] measured as a surrogate of mitochondrial content [51,52]. Specific enzymatic activity was expressed in absolute values as nanomoles of synthesized substrate or consumed product per minute and milligram of protein (nmol/min.mg protein) units and as relative values normalized by CS activity, to relativize the enzymatic activity by mitochondrial content.

### Statistical analysis

Data were analyzed using the IBM SPSS Statistics 21 statistical package. The results were expressed as mean ± standard deviation (SD), median [range] or percentage (n) as appropriate. Normality of data was ascertained with Kolmogorov—Smirnov test. Baseline comparisons between the study groups were performed using the Student *t* test. Categorical variables were analyzed using the Fisher’s exact test. Linear or logistic regression was performed to compare cardiac and mitochondrial parameters among groups adjusted by black ethnicity, low socioeconomic status, smoking during pregnancy and preterm delivery. A subanalysis according to the use of zidovudine during pregnancy was performed using linear regression analysis to test the mitochondrial toxic profile of this specific drug. P-values < 0.05 were considered statistically significant.

## Results

### Study population characteristics

From the initial eligible cohort, two pregnancies from the HIV-exposed group were excluded because of the diagnosis of fetal congenital heart disease (right aortic arch and left hypoplastic heart syndrome), with the final study population including 47 HIV-infected and 47 non-HIV-infected pregnant women. Both populations showed similar baseline epidemiological characteristics (Table 1) with the exception of a higher prevalence of black ethnicity, low socioeconomic status, smoking habit and hepatitis C among the HIV-infected women. Regarding perinatal outcome, HIV pregnancies showed a higher prevalence of preterm delivery and lower birth weight. No mother-to-child transmission of HIV occurred in the present cohort.

**Table 1:** Maternal baseline characteristics and perinatal outcome of the study populations.

	HIV-infected pregnancies (N = 47)	Non HIV-infected pregnancies (N = 47)	p-Value
*Maternal characteristics*			
Age (years)	32.0 ± 5.9	32.4 ± 5.8	0.744
Body mass index (kg/m^2^)	24.7 ± 5.6	24.5 ± 4.8	0.938
Black ethnicity	8 (17)	0	0.009
Nulliparity	20 (42.6)	26 (55.3)	0.144
Low socioeconomic status	17 (36.2)	2 (4.3)	0.001
Smoking during pregnancy	14 (29.8)	5 (10.6)	0.038
Illicit substance use during pregnancy	3 (6.4)	0	0.052
Comorbidity	1 (2.1)	1 (2.1)	-
Hepatitis C infection	6 (12.8)	0	0.026
Hepatitis B infection	2 (4.3)	0	0.495
*Pregnancy and perinatal outcome*			
Use of assisted reproductive technologies	3 (6.4)	2 (4.3)	0.897
Hemoglobin during pregnancy (g/dl)	11.3 ± 1.12	11.4 ± 0.91	0.745
Gestational diabetes	2 (4.3)	2 (4.3)	-
Preeclampsia	3 (6.4)	3 (6.4)	-
Small-for-gestational age	6 (12.8)	3 (6.4)	0.487
Preterm delivery	8 (17.0)	1 (2.1)	0.031
Gestational age at delivery (weeks)	37.6 ± 3.0	39.5 ± 1.6	<0.001
Cesarean section	34 (72.3)	17 (36.2)	0.002
Birth weight (g)	2863 ± 691	3288 ± 459	0.001
5 minutes Apgar score	10 [4–10]	10 [8–10]	0.067
Hospitalization in neonatal intensive care unit	6 (12.8)	1 (2.1)	0.111
Major neonatal morbidity	1 (2.1)	0	0.487
Perinatal mortality	0	0	-

Data are mean ± standard deviation (SD), median [range] or n (percentage).

HIV = Human Immunodeficiency Virus

The immunovirological and therapeutic parameters of the HIV-infected pregnant population are shown in Table 2. Heterosexual transmission was the most common in our population followed by vertical transmission. Eight patients (17%) were diagnosed during pregnancy. Most patients were diagnosed and treated with cART before pregnancy (78.7%). During pregnancy, 46 patients (98%) received cART. The only patient not receiving cART was a late diagnosis after admission for preterm premature rupture of membranes and a subsequent preterm delivery. Regarding the type of NRTI used, 23.4% of the patients were treated with zidovudine. The most common NRTI combinations included: emtricitabine + tenofovir (51%); zidovudine + lamivudine (23.4%) and abacavir + lamivudine (23.4%). The mean CD4 cell count throughout pregnancy was >500 cells/μl. Most patients had an undetectable viral load during pregnancy (70.2% of patients at first trimester and 89.4% at third trimester). Among the five patients with a detectable viral load at delivery, four had less than 1000 copies/ml.

**Table 2:** Immunovirological and therapeutic parameters among the HIV-infected pregnancies.

	n = 47
*HIV transmission*	
Heterosexual	37 (78.7)
Vertical	6 (12.8)
Injection drug use	4 (8.5)
Previous opportunistic infection	13 (27.7)
HIV diagnosis in pregnancy	8 (17.0)
Opportunistic infection during pregnancy	1 (2.1)
Months of HIV infection at delivery	91 [0–324]
*Infection parameters during pregnancy*	
CD4 cell count at first trimester (cells/μl)	550 ± 197
CD4 cell count at delivery (cells/μl)	548 ± 226
Viral load <50 copies/ml at first trimester	33 (70.2)
Viral load <50 copies/ml at delivery	42 (89.4)
Viral load at delivery (patients with detectable viral load copies/ml)	331 [115–6500]
*Antiretroviral treatment characteristics*	
cART before pregnancy	37 (78.7)
Weeks of cART before pregnancy	42 [0–307]
cART during pregnancy	46 (97.9)
Weeks of cART during pregnancy	37.6 [15.2–41]
cART during first trimester pregnancy	34 (72.3)
NRTI during pregnancy	46 (97.9)
Weeks of NRTI during pregnancy	37.3 [12.2–41]
Zidovudine during pregnancy	11 (23.4)
Zidovudine during first trimester	8 (17)
Weeks of zidovudine during pregnancy	36 [20.1–41]
Lamivudine during pregnancy	22 (46.8)
NNRTI during pregnancy	14 (29.8)
Weeks of NNRTI during pregnancy	34.1 [5–40.5]
PI during pregnancy	31 (66)
Weeks of PI during pregnancy	37.2 [10–41]
INI during pregnancy	7 (14.9)

Data are mean ± standard deviation (SD); median [range] or n (percentage) as indicated. HIV = Human Immunodeficiency Virus. cART: Combined antiretroviral treatment; NRTI: Nucleoside reverse transcriptase inhibitors; NNRTI: Non nucleoside reverse transcriptase inhibitors; PI: Protease Inhibitors; INI: Integrase inhibitors.

### Fetal cardiovascular results

At the time of fetal echocardiography, the estimated fetal weight was similar between groups (HIV 1725 g (676) vs. non-HIV 1862 g (537), P = 0.297) with no signs of placental insufficiency (cerebroplacental ratio: HIV 2.05 (0.57) vs. non-HIV 2.33 (0.84), P = 0.071). Fetuses from HIV-infected mothers showed larger and hypertrophic hearts (increased cardio-thoracic ratio and myocardial wall thicknesses) as compared to non-HIV-exposed fetuses. Moreover, 21% of fetuses from HIV-infected pregnancies presented mild pericardial effusion (2–4 mm). While the ejection fraction was preserved, fetuses from HIV-pregnancies also showed signs of decreased longitudinal motion (lower mitral S’) and impaired diastolic function (increased left isovolumic relaxation time) as compared to non-HIV-infected pregnancies. Most fetal cardiac changes remained statistically significant after adjustment by black ethnicity, low socioeconomic status, smoking during pregnancy and maternal hepatitis C infection (Table 3). Cord blood plasmatic BNP levels were significantly increased in the HIV cohort as compared to the non-HIV cohort (HIV 26.5 pg/ml (19.1) vs. non-HIV 19.3 pg/ml (12.3), p = 0.04), although statistical significance was lost after adjustment for maternal black ethnicity, smoking during pregnancy and preterm delivery (adjusted p = 0.85).

**Table 3:** Fetal echocardiographic results among the study groups.

	HIV-infected pregnancies (n = 47)	Non HIV-infected pregnancies (n = 47)	Adjusted p-Value*
*Gestational age at scan (weeks)*	30.9 ± 2.8	31.2 ± 3.2	0.189
*Cardiac morphology*			
Cardio-thoracic ratio	0.30 ± 0.05	0.27 ± 0.03	0.029
Pericardial effusion	21 (44.7)	1 (2.1)	<0.001
Left free wall myocardial thickness (mm)	3.21 ± 0.81	2.72 ± 0.42	0.012
Septal wall myocardial thickness (mm)	3.80 ± 0.88	3.59 ± 0.63	0.096
Right free wall myocardial thickness (mm)	3.13 ± 0.65	2.81 ± 0.42	0.023
*Cardiac function*			
Left ejection fraction (%)	72.93 ± 12.53	70.59 ± 8.79	0.575
Mitral ring displacement (mm)	5.19 ± 0.94	5.65 ± 1.09	0.183
Tricuspid ring displacement (mm)	7.24 ± 1.23	7.16 ± 1.47	0.797
Mitral S’ (cm/s)	6.76 ± 1.19	7.27 ±0.81	0.007
Tricuspid S’ (cm/s)	7.52 ± 1.28	7.72 ± 1.15	0.201
Mitral E’ (cm/s)	6.93 ± 0.94	7.05 ± 1.09	0.583
Tricuspid E’ (cm/s)	8.15 ± 1.26	7.51 ± 1.19	0.015
Isovolumic contraction time (ms)	40.9 ± 8.16	36.9 ± 8.68	0.028
Isovolumic relaxation time (ms)	52.2 ± 8.85	42.5 ± 7.30	<0.001
Ejection time (ms)	163 ± 17.55	171 ± 13.72	0.025
Myocardial performance index	0.58 ± 0.13	0.47 ± 0.09	<0.001

Data are mean ± standard deviation or n (percentage).

HIV = Human Immunodeficiency Virus. S’ = systolic annular peak velocity. E’ = annular peak velocity in early diastole.

* p-value calculated by linear or logistic regression adjusted by black ethnicity, low socioeconomic status, smoking during pregnancy and maternal hepatitis C infection.

**Fig 1:**
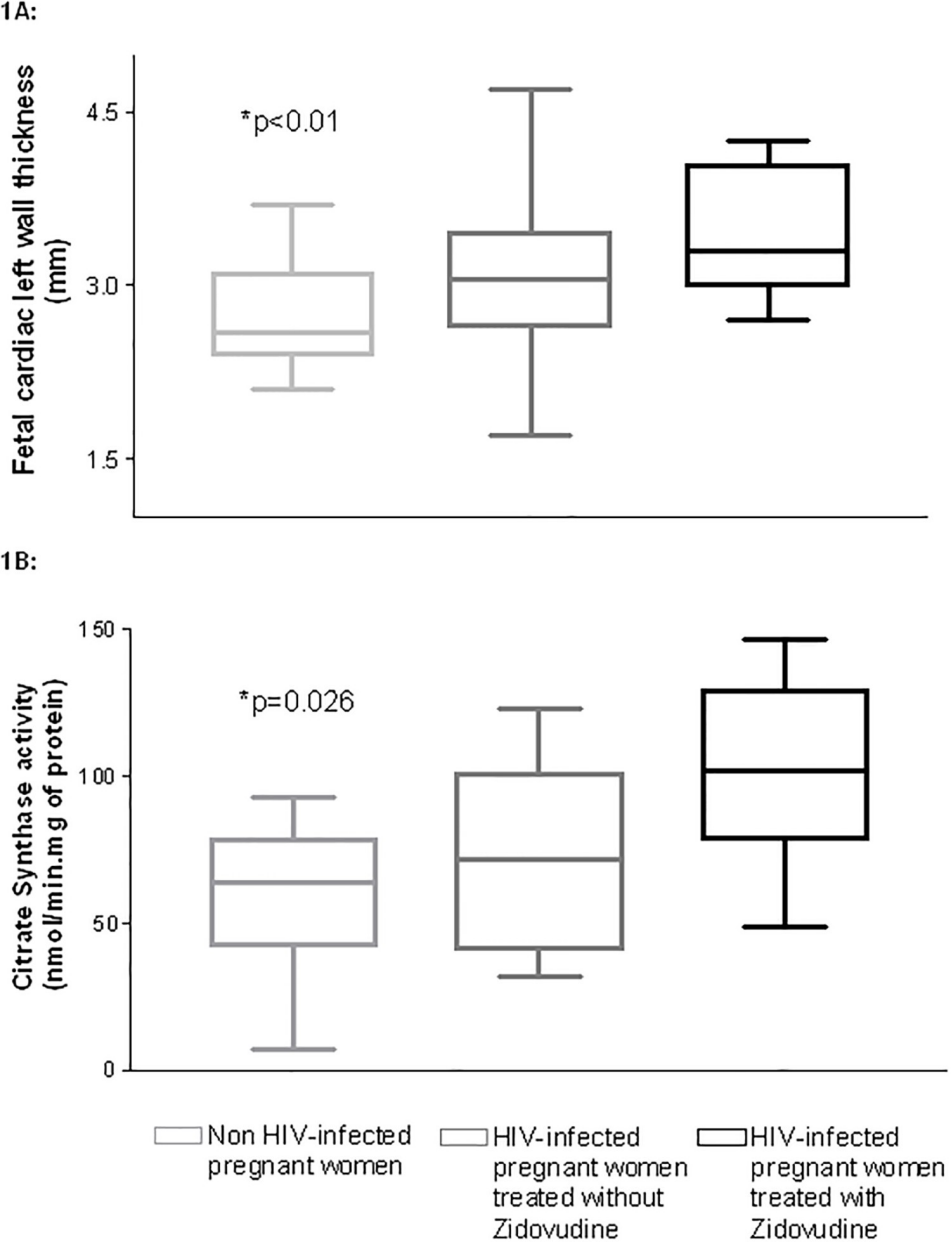
Results on fetal myocardial thickness (A) and mitochondrial content -citrate synthase activity- (B) according to the maternal use of zidovudine. Box and whiskers graph of fetal myocardial left wall thickness (1A) and mitochondrial content -citrate synthase activity- (1B) among non HIV-infected and HIV-infected subdivided according to the use of zidovudine during pregnancy. A significant trend of increased myocardial thickness and mitochondrial content was observed among the categories. p-value was calculated by ANOVA test among the three categories. Boxes in the graphic represent 25th, 50th (band inside the box), and 75th centile values. Whiskers express dispersion (1.5*interquartile range).

A subanalysis to investigate the impact of maternal cART on the fetal heart was further performed by subdividing the HIV-infected population according to the use of zidovudine during pregnancy. No differences were observed in maternal baseline characteristics, in perinatal outcomes or HIV-infection parameters among the study subgroups. Notably, a significant linear tendency to worse cardiovascular results among the subgroups was observed, with the subgroup receiving zidovudine showing the worst results. There was a significant trend to progressively thicker fetal myocardial wall thicknesses among the subgroups (left free wall thickness: non-HIV 2.61 mm (0.43) vs. HIV treated without zidovudine 3.10 mm (0.86) vs. HIV treated with zidovudine 3.34 mm (0.54), p<0.01; and septal wall thickness: non-HIV 3.40 mm (0.59) vs. HIV treated without zidovudine 3.65 mm (0.87) vs. HIV treated with zidovudine 3.95 mm (0.71), p = 0.041) (Fig 1A).

In addition, IRT was significantly longer across groups (non-HIV 41.18 ms (7.9) vs. HIV treated without zidovudine 51.55 ms (9.19) vs. HIV treated with zidovudine 59.21 ms (9.35); p<0.01).

### Mitochondrial results

The cord blood mitochondrial content, represented by CS activity, was significantly increased in CBMC from HEU fetuses with respect to non-HIV-exposed fetuses (Table 4). Mitochondrial DNA content and function were preserved with similar levels of mtDNA and COX enzymatic activity. All the mitochondrial parameters studied in placental tissue were similar between the study populations.

**Table 4:** Cord blood and placental mitochondrial results among the study groups. Data are mean ± standard deviation.

	HIV-infected pregnancies (n = 47)	Non HIV-infected pregnancies (n = 47)	Adjusted p-Value*
*Cord blood*			
mtDNA (12SrRNA/RNAseP)	95.2 ± 33.0	90.4 ± 27.6	0.654
mtDNA/CS(12SrRNA/RNAseP normalized by CS)	3.81 ± 2.29	4.20 ± 3.53	0.433
COX activity (nmol/min.mg of protein)	33.2 ± 20.2	30.8 ± 18.9	0.295
CS activity (nmol/min.mg of protein)	82.9 ± 40.5	56.7 ± 28.4	0.007
COX/CS (nmol/min.mg of protein normalized by CS)	0.44 ± 0.27	0.57 ± 0.29	0.530
*Placenta*			
mtDNA (12SrRNA/RNAseP)	94.7 ± 19.1	91.5 ± 26.7	0.665
mtDNA/CS (12SrRNA/RNAseP ratio normalized by CS)	3.06 ± 1.22	2.95 ± 1.43	0.945
COX activity (nmol/min.mg of protein)	16.7 ± 6.02	17.9 ± 6.78	0.799
CS activity (nmol/min.mg of protein)	34.8 ± 13.1	33.7 ± 11.3	0.759
COX/CS (nmol/min.mg of protein normalized by CS)	0.50 ± 0.15	0.58 ± 0.24	0.353

mtDNA: mitochondrial DNA. CS: Citrate Synthase. COX: cytochrome c oxidase.

* P-value calculated by linear regression adjusted by black ethnicity, low socioeconomic status, smoking during pregnancy, maternal hepatitis C infection, C-section and birth weight.

The subanalysis according to the use of zidovudine (Fig 1B) revealed a significant linear tendency to progressively higher CS activity in HIV-infected pregnancies using zidovudine as compared to HIV-infected pregnancies not using zidovudine and non-infected pregnancies (109.79 nmol/min.mg of protein (50.42) vs. 70.28 nmol/min.mg of protein (30.68) vs. 56.7 nmol/min.mg of protein (28.4) respectively; p = 0.026).

We found no significant correlation between mitochondrial data and maternal immunological status, fetal cardiovascular results or perinatal outcome.

## Discussion

In the present study, we found signs of increased fetal myocardial mass and mitochondrial content most likely enabling a preserved cardiac ejection fraction and mitochondrial function in HEU fetuses exposed to maternal cART. Both cardiovascular and mitochondrial mass-related parameters were significantly associated with zidovudine exposure.

The present study confirms previous results suggesting the presence of fetal cardiac remodeling in HIV-infected pregnancies, mainly in the form of fetal myocardial hypertrophy. HIV-exposed fetuses showed larger hearts with a higher prevalence of pericardial effusion, signs of increased myocardial mass, reduced systolic motion and impaired relaxation with preserved ejection fraction. These results are in agreement with previous literature in HEU fetuses [53] and suggest that fetal cardiac hypertrophy compensates for myocardial toxicity in the context of HIV infection and cART exposure. Indeed, the present study also confirms previous data suggesting a significant association between fetal myocardial hypertrophy and the use of zidovudine during pregnancy [11].

This study also aimed to assess fetal and placental mitochondrial function in HIV pregnancies. We observed an increased CS activity in the fetal CBMC of the HIV-exposed group, with a similar mtDNA content and COX enzymatic activity. Citrate synthase activity is one of the biomarkers exhibiting the strongest association with mitochondrial content [52]. No differences were detected in any of the mitochondrial parameters in placental tissue between groups. Our results in cord blood may reflect impaired fetal mitochondrial function, which triggers a compensatory mechanism consisting of increasing the number of mitochondria to preserve a normal mitochondrial activity, as suggested in previous studies [54]. Interestingly, these results are concordant with the fetal cardiac findings. The hypertrophic pattern observed in the fetal echocardiography, with a higher myocardial mass, might enable a preserved global systolic function, reflected by a normal fetal ejection fraction. In addition, the CS activity in fetal CBMC, reflecting the mitochondrial content in fetal samples, similar to the hypertrophic phenotype described in the fetal cardiac evaluation, showed a significant positive correlation with the maternal use of zidovudine during pregnancy. Comparison with previous data on mitochondrial toxicity in HEU children is difficult as the results of the different studies available are controversial and vary depending on the study population. Indeed, we found no changes in mtDNA while previous studies suggested differences in mtDNA content or mitochondrial respiratory chain dysfunction in HEU children [19,21,24,54,55]. Differences in the cART regimens during pregnancy among studies could also be a potential explanation for the different results observed in our cohort. Most studies reporting depletion of mtDNA and mitochondrial activity included a large proportion of pregnancies receiving zidovudine, while in our cohort only 23.4% of the HIV pregnant women were treated with zidovudine. In fact, zidovudine is recognized as one of the most toxic drugs for mitochondria due to its capacity to inhibit mitochondrial DNA polymerase gamma [56]. On the other hand, cART regimens during pregnancy have evolved over time according to updated guidelines in which zidovudine is no longer the first treatment option during pregnancy since it requires complex dosing and is associated with higher rates of adverse effects [32,33]. Therefore, we hypothesized that populations under relatively more toxic cART regimens would present impaired mitochondrial function, while other populations such as that in the present study receiving less toxic therapies could compensate and maintain function by increasing mitochondrial mass.

The present study has some strengths and limitations that merit comment. One of the main strengths is that this is the first work simultaneously assessing fetal cardiac morphology and function together with cord blood mitochondrial toxicity parameters in HIV-pregnancies. The comprehensive fetal cardiac evaluation demonstrated significant changes in cardiac structure and function in HIV-exposed fetuses. Moreover, the present study proposes a possible pathogenic mechanism involved in fetal cardiac abnormalities, with a new pattern of mitochondrial impairment in HEU fetuses under cART related to the use of zidovudine. We also acknowledge some limitations. Firstly, the relatively small sample size of the study could have hampered some statistical differences and limited further subanalysis among the HIV population. For instance, the correlation between fetal cardiac and mitochondrial results may have been hampered by the limited number of patients treated with zidovudine. Secondly, we acknowledge the potential influence of confounders such as maternal smoking status, concomitant infections or other pregnancy complications. However, a multivariate analysis was conducted in order to minimize a potential confounding effect. Regarding mitochondrial studies, despite platelet contamination cannot be totally discarded in the samples studied, its minimal representation, abrogates for negligible effects. Finally, mitochondrial function was studied in fetal CBMC and in placental homogenates, which may not reflect the real mitochondrial toxicity in the fetal heart. Therefore, future experimental studies directly evaluating fetal cardiac mitochondrial toxicity in animal models are needed.

In conclusion, in the present cohort we confirmed the presence of fetal cardiac hypertrophy and further report signs of increased mitochondrial number in HEU fetuses, being both associated with maternal zidovudine treatment during pregnancy. These results highlight the relevance of prenatal studies evaluating the impact of different cART regimens on HEU children in order to identify the safest options to be incorporated in the clinical management of these pregnancies. It also reinforces the concept of fetal programming that defines prenatal life as a critical period for fetal organ development during which any environmental change might affect and have an impact on future health. Further studies are warranted to evaluate the potential long-term impact on cardiovascular health in the constantly evolving HEU cohorts under different cART regimens.
